# Functional Interplay Between Murine Leukemia Virus Glycogag, Serinc5, and Surface Glycoprotein Governs Virus Entry, with Opposite Effects on Gammaretroviral and Ebolavirus Glycoproteins

**DOI:** 10.1128/mBio.01985-16

**Published:** 2016-11-22

**Authors:** Yadvinder S. Ahi, Shu Zhang, Yashna Thappeta, Audrey Denman, Amin Feizpour, Suryaram Gummuluru, Bjoern Reinhard, Delphine Muriaux, Matthew J. Fivash, Alan Rein

**Affiliations:** a HIV Dynamics and Replication Program, National Cancer Institute, Frederick, Maryland, USA; b Department of Chemistry and The Photonics Center, Boston University, Boston, Massachusetts, USA; c Department of Microbiology, Boston University School of Medicine, Boston, Massachusetts, USA; d Centre d’Etudes d’Agents Pathogènes et Biotechnologies pour la Santés, CNRS UMR 5236, Montpellier, France; e Data Management Services, National Cancer Institute at Frederick, Frederick, Maryland, USA

## Abstract

Gammaretroviruses, such as murine leukemia viruses (MLVs), encode, in addition to the canonical Gag, Pol, and Env proteins that will form progeny virus particles, a protein called “glycogag” (glycosylated Gag). MLV glycogag contains the entire Gag sequence plus an 88-residue N-terminal extension. It has recently been reported that glycogag, like the Nef protein of HIV-1, counteracts the antiviral effects of the cellular protein Serinc5. We have found, in agreement with prior work, that glycogag strongly enhances the infectivity of MLVs with some Env proteins but not those with others. In contrast, however, glycogag was detrimental to MLVs carrying Ebolavirus glycoprotein. Glycogag could be replaced, with respect to viral infectivity, by the unrelated S2 protein of equine infectious anemia virus. We devised an assay for viral entry in which virus particles deliver the Cre recombinase into cells, leading to the expression of a reporter. Data from this assay showed that both the positive and the negative effects of glycogag and S2 upon MLV infectivity are exerted at the level of virus entry. Moreover, transfection of the virus-producing cells with a Serinc5 expression plasmid reduced the infectivity and entry capability of MLV carrying xenotropic MLV Env, particularly in the absence of glycogag. Conversely, Serinc5 expression abrogated the negative effects of glycogag upon the infectivity and entry capability of MLV carrying Ebolavirus glycoprotein. As Serinc5 may influence cellular phospholipid metabolism, it seems possible that all of these effects on virus entry derive from changes in the lipid composition of viral membranes.

## IMPORTANCE

Many murine leukemia viruses (MLVs) encode a protein called “glycogag.” The function of glycogag is not fully understood, but it can assist HIV-1 replication in the absence of the HIV-1 protein Nef under some circumstances. In turn, Nef counteracts the cellular protein Serinc5. Glycogag enhances the infectivity of MLVs with some but not all MLV Env proteins (which mediate viral entry into the host cell upon binding to cell surface receptors). We now report that glycogag acts by enhancing viral entry and that, like Nef, glycogag antagonizes Serinc5. Surprisingly, the effects of glycogag and Serinc5 upon the entry and infectivity of MLV particles carrying an Ebolavirus glycoprotein are the opposite of those observed with the MLV Env proteins. The unrelated S2 protein of equine infectious anemia virus (EIAV) is functionally analogous to glycogag in our experiments. Thus, three retroviruses (HIV-1, MLV, and EIAV) have independently evolved accessory proteins that counteract Serinc5.

## INTRODUCTION

Gammaretroviruses, such as murine leukemia viruses (MLVs), are frequently considered prototypical “simple retroviruses,” encoding only the canonical Gag, Pol, and Env proteins needed to assemble infectious progeny virus particles. However, many gammaretroviruses encode an additional protein called “glycogag” (glycosylated Gag [also “gGag”]). In MLV, this protein is identical in primary sequence to Gag except that it contains 88 additional residues at its N terminus (1). The N-terminal extension includes a signal sequence, and the protein is believed to be processed through the secretory pathway and transported to the cell surface. It is a type II integral membrane protein, with its N terminus in the cytoplasm and its C terminus outside; it is ultimately cleaved once by an unknown cellular protease, and the C-terminal fragment is released into the medium (2–4).

The function of glycogag is not understood. Reportedly, it is more important for MLV replication in mice than in cell culture (5). It has also been said to improve the quality and quantity of virus assembly and release (6), enhance the stability of the mature viral capsid (7), direct virus production to lipid rafts in virus-producing cells (8), enhance viral pathogenicity (5, 9), and protect MLV from inactivation by the restriction factor mouse APOBEC3 (mA3) (10). Remarkably, it has also been reported to complement a Nef defect in HIV-1 (11).

The analysis of glycogag function has been complicated by the fact that the same sequence in viral RNA, the *gag* gene, codes for both the Gag protein and the majority of glycogag. We have developed reagents for the independent expression of tagged versions of both proteins. These reagents have enabled us to follow both proteins in the cell and to assess the contributions of glycogag to steps in the viral replication cycle. We now report that glycogag has a profound effect upon the ability of MLV to enter the host cell. However, the effect of glycogag is critically dependent upon the identity of the envelope on the virus. Unexpectedly, we find that the effects of glycogag upon MLV carrying Ebolavirus glycoprotein (GP) are the opposite of its effects on MLV with xenotropic or amphotropic MLV Env glycoprotein. The reasons for this are not yet clear.

## RESULTS

### Independent expression of Gag and glycogag.

**FIG 1:**
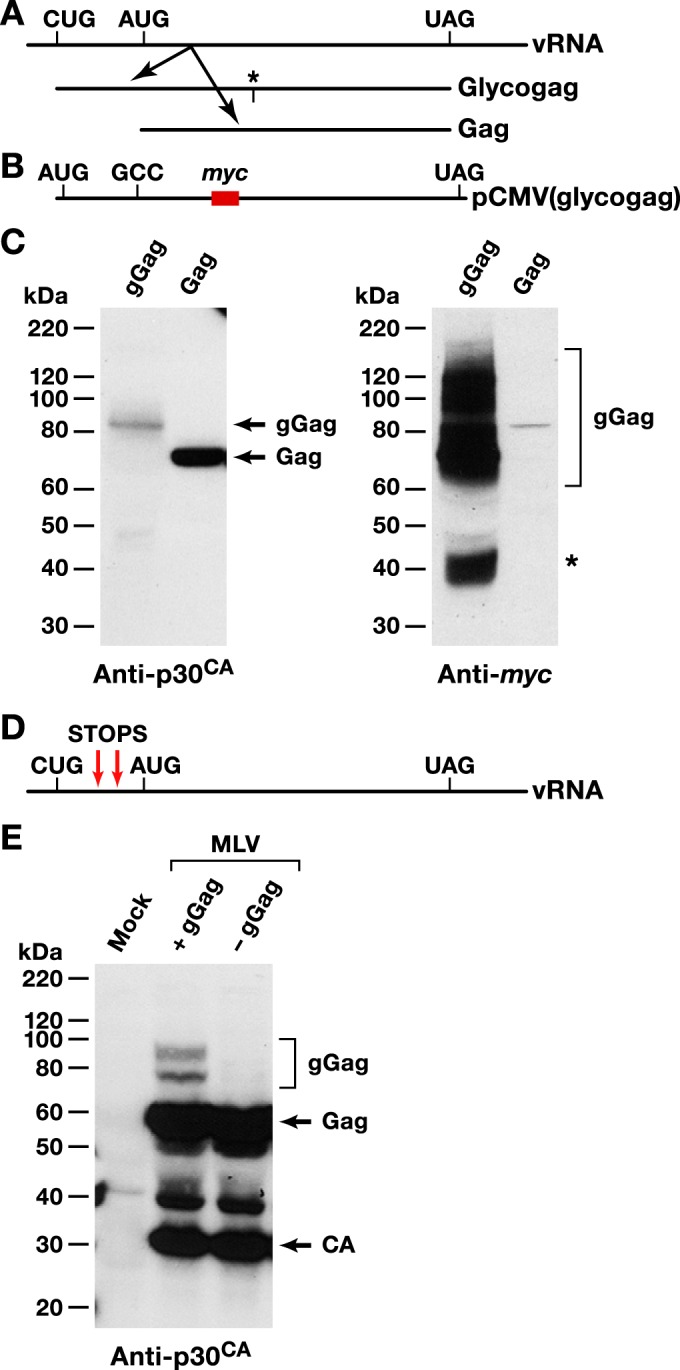
Glycogag expression plasmid and Glycogag-negative MLV clone. (A) Schematic of translation of glycogag and Gag from the viral genome. *, cleavage site in glycogag. Precise location of cleavage site is not known. (B) Schematic of pCMV(glycogag), the glycogag expression plasmid. (C) Lysates of cells transfected with pCMV(glycogag) or Gag expression plasmids were probed with anti-p30^CA^ or anti-Myc antibodies. gGag and Gag bands are indicated. *, cleavage product of gGag. (D) Schematic of glycogag-negative Moloney MLV clone. (E) Lysates of cells transfected with wild-type (+gGag) or glycogag-negative (−gGag) Moloney MLV clones or of mock-transfected cells were probed with anti-p30^CA^ antibody at 48 h posttransfection. gGag, Gag, and capsid (CA) bands are indicated.

In order to obtain clear information on the fate and function of glycogag despite its close relationship to Gag, it was essential to control the expression of both proteins and to be able to specifically detect each of them. In the natural setting, glycogag is expressed from a CUG codon in the viral RNA, 88 codons upstream from the normal Gag AUG initiator (Fig. 1A) (1). To generate a glycogag expression plasmid, we began with a codon-optimized expression plasmid for Gag and first inserted the 88 codons on the 5′ side of the AUG (the sequence of the Gag protein here was that of xenotropic murine leukemia virus-related virus [XMRV], a gammaretrovirus very similar to Moloney MLV). We then replaced the CUG glycogag initiator with an AUG. To prevent leaky scanning, leading to Gag synthesis from the plasmid, we also replaced the Gag AUG with the alanine codon GCC (Fig. 1B). Finally, we inserted sequences for a *myc* epitope tag into the p12 region of the plasmid, at a site previously shown to be tolerant of insertions (12–14). As shown by the results in Fig. 1C, this plasmid, designated pCMV(glycogag), directs the synthesis of glycogag but not that of Gag; the glycogag can be detected with antiserum against either p30^CA^ or Myc.

We also wished to create an infectious MLV clone that did not express glycogag. The CUG triplet at which glycogag is initiated is part of one of the two stems in viral RNA with GACG in the loop at the apex of the stem. These conserved stem-loops (15) are essential for the virus, as they are the strongest junctions between the monomers in dimeric viral RNA (16). We therefore blocked glycogag synthesis without disturbing the CUG by introducing two in-frame stop codons between the CUG and the Gag AUG of a proviral clone (Fig. 1D). As shown by the results in Fig. 1E, the lysates of cells producing the mutant virus lack the gGag-specific bands seen in the lysates of cells producing wild-type virus.

### Trafficking of glycogag.

**FIG 2:**
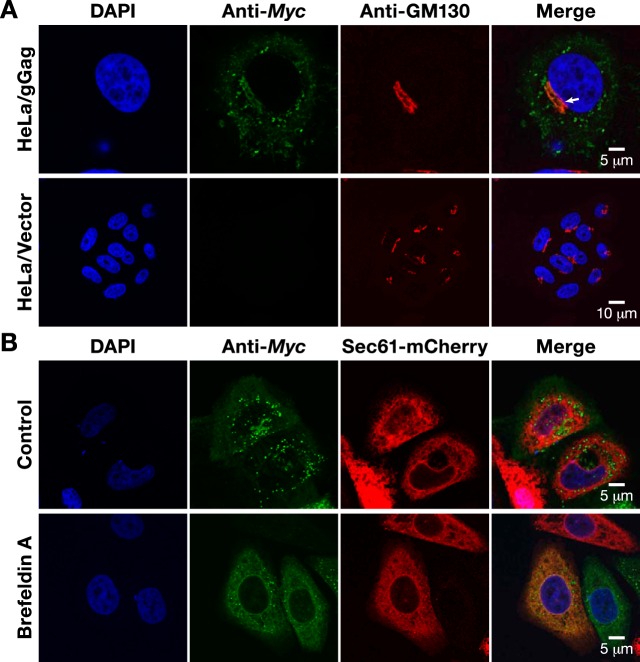
Glycogag traffics through endoplasmic reticulum and Golgi apparatus. (A) Confocal microscopy of HeLa/gGag cells and control HeLa/vector cells cultured in the presence of 10-ng/ml doxycycline for 24 h and stained with anti-Myc antibody for gGag and anti-GM130 for Golgi apparatus. (B) Confocal microscopy of HeLa/gGag cells transiently expressing Sec61-mCherry fusion protein cultured in the presence of 10-ng/ml doxycycline for 24 h. The cells were treated with 200-ng/ml brefeldin A for 3 h, followed by fixation and immunostaining with anti-Myc antibody (for detection of gGag). DAPI was used for staining nuclei. The arrow in panel A indicates localization of gGag in Golgi apparatus.

We expressed Myc-tagged glycogag under doxycycline control in HeLa cells, using the *piggyBac* transposon system described by Li et al. (17). We determined its intracellular location by immunostaining against the Myc tag. The staining was largely punctate within the cytoplasm, but there was also a significant concentration at a perinuclear location. As the glycogag at this site colocalized with GM130, a Golgi marker, this site is evidently the Golgi apparatus (Fig. 2A). Furthermore, we noted that in cells treated with brefeldin A, which arrests trafficking from the endoplasmic reticulum (ER) to the Golgi apparatus, glycogag colocalized with the endoplasmic reticulum (ER) marker Sec61 (Fig. 2B). We conclude that, like most cell surface proteins, glycogag is produced in the rough ER and traffics through the Golgi apparatus.

We also inserted the Flag epitope tag into the p12-coding region of the codon-optimized XMRV Gag expression plasmid. We coexpressed this Flag-tagged Gag together with the Myc-tagged glycogag and stained for the epitope tags in the respective proteins. Glycogag did not appear to colocalize with Gag to any significant extent (see Fig. S1 in the supplemental material).

### Effects of glycogag on virion infectivity.

**FIG 3:**
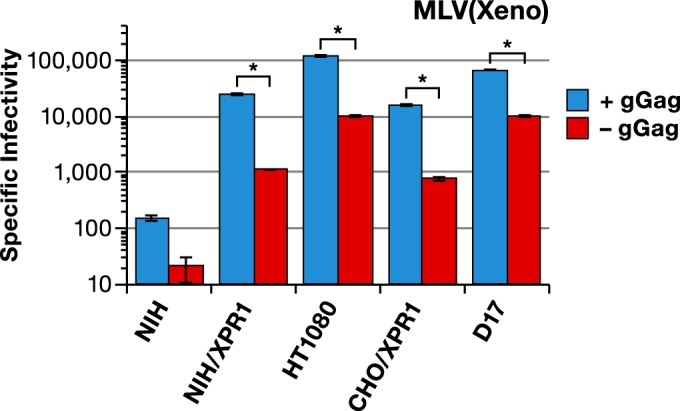
Effect of gGag on infectivity of MLV(Xeno). Specific infectivities (luciferase activity units divided by relative amounts of p30^CA^) of MLV(Xeno) with wild-type Gag-Pol (blue bars) or mutant Gag-Pol lacking gGag (red bars) produced in 293T cells and assayed on the indicated cell lines. NIH, NIH/3T3 mouse cells; hXPR1, human XPR1; *, *P* < 0.0001.

Several years ago, Pizzato reported that the specific infectivity of MLV particles produced in the presence of glycogag was significantly higher than in its absence (11). Remarkably, this effect of glycogag seemed to depend upon (among other variables) the type of Env present in the virus. We have extended these findings in several ways. First, we tested the possibility that the identity of the target cell, as well as that of the Env, might influence the glycogag requirement. We measured the specific infectivities of MLV particles with xenotropic (Xeno) Env proteins [designated “MLV(Xeno)” below], with or without glycogag, on four different host cells, all displaying the XPR1 xenotropic MLV receptor. The virus particles were produced by cotransfecting either a full-length proviral clone whose Env-coding region had been destroyed by a frameshift or the glycogag-negative version of this clone (Fig. 1D), together with a xenotropic Env expression clone. As shown by the results in Fig. 3, glycogag increased the specific infectivity of the virus roughly 10-fold, whether it was measured on human (HT1080) or dog (D17) cells, which express the receptor naturally, or on mouse (NIH3T3) or hamster (CHO) cells that had been engineered to express human XPR1. Thus, the deficit in the infectivity of the virus lacking glycogag is evidently independent of the species of cells on which it is assayed.

**FIG 4:**
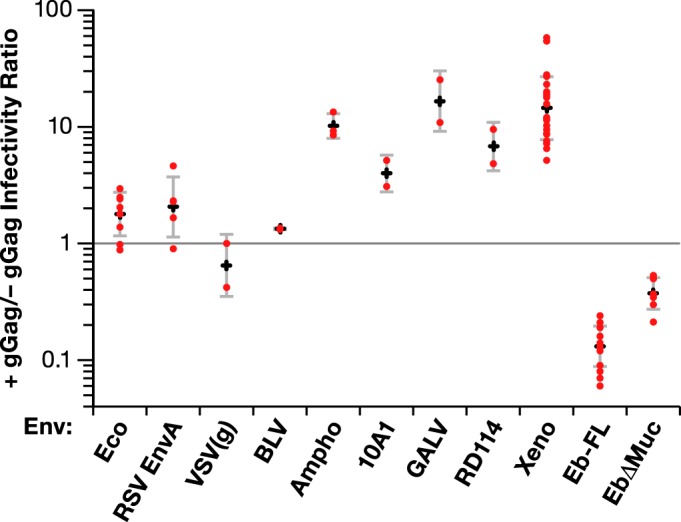
Effect of gGag on MLV infectivity is determined by Env protein. Specific infectivities of MLV with indicated Env glycoproteins produced in the presence or absence of gGag. Viruses were produced with wild-type or gGag-deficient Gag-Pol together with the indicated Env expression clones. The *x* axis shows the type of Env glycoprotein on the virus. For each Env, the red dots represent the ratios of specific infectivities of virus with gGag to virus without gGag in individual experiments. The plus signs show the geometric means of these values, and the bars at the top and bottom of each vertical line show associated 95% confidence intervals for each Env. Eb-FL, full-length Ebola glycoprotein; EbΔMuc, Ebola glycoprotein with deletion of mucinlike domain. The target cell line used for viruses with VSV(g), Ampho (amphotropic), 10A1, GALV, RD114, Xeno (xenotropic), Eb-FL, and EbΔMuc glycoproteins was HT1080, the target cell line used for viruses with Eco (ecotropic) and BLV glycoprotein was HT1080/mCAT1, and the target cell line used for viruses with RSV Env A was D17 cells expressing subgroup A receptor.

MLV Envs are polymorphic, and different viral isolates use different cell surface receptors in infection. We tested the glycogag requirement for MLV particles carrying a wide variety of Env proteins. The effects of glycogag on the specific infectivities are presented in Fig. 4 as the ratio of the specific infectivity of the virus produced with glycogag to that produced in the absence of glycogag. We found that there was a strong glycogag requirement for viruses carrying not only amphotropic (Ampho) or xenotropic Env (as reported by Pizzato [11]) but also with Env from 10A1 MLV, a highly leukemogenic recombinant derived from amphotropic MLV that uses both the amphotropic receptor SLC20A2 and a second, related receptor, SLC20A1, for entry into cells (18, 19). In contrast, as shown by Pizzato (11), the specific infectivity of virus with the Moloney MLV ecotropic Env [MLV(Eco) Env] and vesicular stomatitis virus glycoprotein [VSV(g)] is not significantly affected by the presence of glycogag (Fig. 4).

We also tested the glycogag requirement with MLVs using several heterologous Env proteins (Fig. 4). The glycogag requirement was also seen with the Env of the gammaretrovirus gibbon ape leukemia virus (GALV). We saw a similar result with the Env of the feline gammaretrovirus RD114; this was of interest because these two Env proteins determine differential sensitivity to a recently described cellular restriction system (20). In contrast, glycogag did not significantly affect the infectivity of MLV with the subgroup A Env protein of the alpharetrovirus Rous sarcoma virus (RSV) or the deltaretrovirus bovine leukemia virus (BLV).

The effects of glycogag on the specific infectivity of MLV carrying the Ebolavirus GP were also tested. Remarkably, as shown by the results in Fig. 4, in this case virus lacking glycogag had a significantly higher specific infectivity than that containing glycogag. The use of Ebolavirus GP with a deletion of the mucinlike domain (EbΔMuc) has been reported to yield MLV pseudotypes with higher titers than those obtained with full-length Ebolavirus GP (21, 22). Therefore, we also tested the effect of glycogag upon MLV particles with this deletion mutant of Ebolavirus GP. As shown by the results in Fig. 4, glycogag also reduced the specific infectivity of these particles, although this effect was somewhat smaller than that with the full-length Ebolavirus.

**FIG 5:**
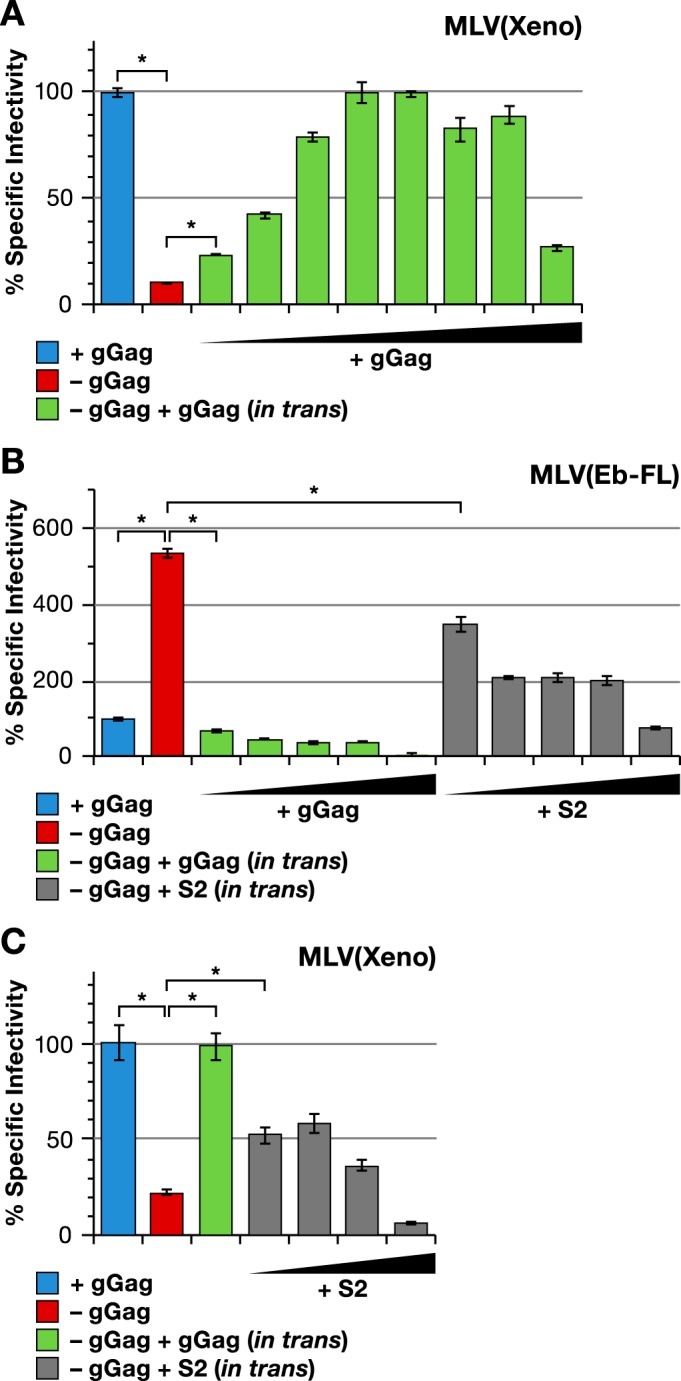
Effect of MLV gGag and EIAV S2 expressed in *trans* on infectivity of MLV(Xeno) and MLV(Ebola). (A) Specific infectivities of MLV(Xeno) with wild-type Gag-Pol (blue bar) or mutant Gag-Pol lacking gGag (red bar), and MLV(Xeno) with mutant Gag-Pol cotransfected with increasing amounts of pCMV(glycogag) (green bars). (B and C) Specific infectivity of MLV(Eb-FL) (B) and MLV(Xeno) (C) with wild-type Gag-Pol (blue bar) or with mutant Gag-Pol lacking gGag (red bar), together with pCMV(glycogag) or S2 expression plasmid. The pCMV(glycogag)/Gag-Pol plasmid ratios used were increased by threefold increments from 1:6,561 to 1:3 in the experiment whose results are shown in panel A and from 1:243 to 1:3 in the experiment whose results are shown in panel B; in the experiment whose results are shown in panel C, the ratio used was 1:27. The S2/gag-Pol plasmid ratios used were increased by threefold increments from 1:243 to 1:3 in the experiment whose results are shown in panel B and from 1:81 to 1:3 in the experiment whose results are shown in panel C. The target cell line used in these experiments was HT1080/mCAT1. *, *P* < 0.0001.

Using the pCMV(glycogag) plasmid described above, we also attempted to complement in *trans* the infectivity defect in glycogag-negative MLV bearing xenotropic Env. As shown by the results in Fig. 5A, cotransfection of the expression plasmid largely restored the specific infectivity of the virus. Titration of the expression plasmid revealed that the optimal stoichiometry for virus rescue was approximately 1 glycogag plasmid to approximately 100 Gag-Pol plasmids, with the specific infectivity of the virus declining at higher, as well as lower, plasmid ratios. We also added pCMV(glycogag) to the plasmids used to produce MLV particles with Ebolavirus GP [MLV(Ebola)] and found (Fig. 5B, green bars) that it substantially reduced the specific infectivity of the virus.

### Complementation of glycogag defect with EIAV S2 protein.

Another retroviral protein of unknown function is the S2 protein of equine infectious anemia virus (EIAV). S2 is essential for the pathogenicity of EIAV (23). It is only 65 to 68 residues in length and has no notable sequence resemblance to either Nef or glycogag. However, it has recently been found to complement a Nef defect in HIV-1 (71). It was therefore of interest to determine whether S2 could replace glycogag in MLV infections. As shown by the results in Fig. 5B, EIAV S2, like glycogag, reduces the infectivity of MLV carrying Ebolavirus GP. In contrast, it substantially rescues the infectivity of glycogag-negative MLV carrying xenotropic Env (Fig. 5C).

### Y36A mutation reduces glycogag activity.

**FIG 6:**
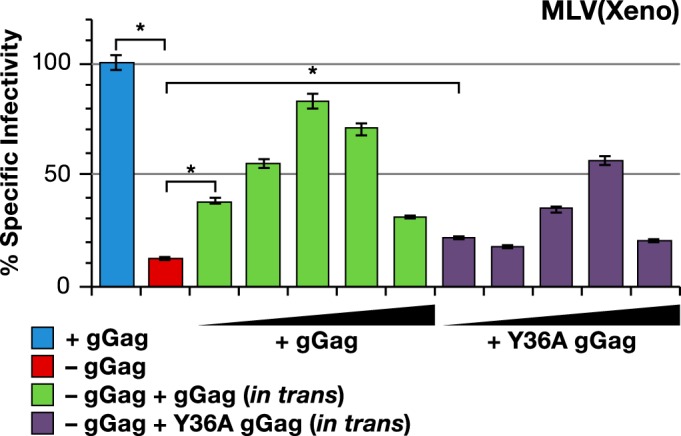
Y36A mutant of gGag is partially active in enhancing MLV(Xeno) infectivity. Specific infectivity of MLV(Xeno) with wild-type Gag-Pol (blue bar) or with mutant Gag-Pol lacking gGag (red bar), and MLV(Xeno) with mutant Gag-Pol cotransfected with increasing amounts of wild-type (green bars) or Y36A mutant gGag (purple bars) pCMV(glycogag). The wild-type or Y36A mutant gGag/Gag-Pol plasmid ratios used were increased by threefold increments from 1:243 to 1:3. The target cell line used in these experiments was HT1080/mCAT1. *, *P* < 0.0001.

As mentioned above, glycogag has the ability to complement the Nef defect in Nef^−^ HIV-1. Usami et al. reported that this activity is reduced or lost if tyrosine 36 (in the glycogag-specific region of glycogag) is replaced with alanine (Y36A mutation) (24). We introduced the Y36A mutation into pCMV(glycogag) and tested the ability of the mutant to enhance MLV(Xeno) infectivity. The results in Fig 6 show the effects of dilution series of wild-type and Y36A glycogag plasmids upon the specific infectivity of MLV(Xeno) lacking glycogag. While it is difficult to compare the two titrations precisely, the data show clearly that the mutant glycogag retains partial activity in this assay. As a 1:9 dilution of the Y36A plasmid (Fig 6, fourth purple bar) had an effect similar to the 1:81 dilution of the wild-type plasmid (Fig 6, second green bar), perhaps the mutant is ~1/10 as active as wild-type glycogag.

### Glycogag does not affect annexin V binding to MLV particles.

It was recently reported that glycogag, like HIV-1 Nef, counteracts the effects of the cellular protein Serinc5 (25, 26). While little is known about Serinc5, it seems likely that it affects phospholipid metabolism (27). In turn, this raises the possibility that glycogag influences the lipid composition of virions. As one approach to this question, we quantitated the binding of annexin V to viral surfaces (28): annexin V is known to bind phosphatidylserine (PS) but may also bind phosphatidylethanolamine (PE) (29). However, as shown by the results in Fig. S2 in the supplemental material, the presence of glycogag had no detectable effect on the binding of annexin V to MLV particles carrying xenotropic or ecotropic Env (see Fig. S2A) or full-length Ebolavirus glycoprotein (see Fig. S2B).

### Lack of protection against mouse APOBEC3 restriction.

Among the functions that have been attributed to glycogag is protection of MLVs against restriction by mA3 (7, 10, 30, 31). We have tested for this activity in our experimental system. MLV particles carrying xenotropic Env were produced with and without glycogag and with varying doses of mA3 expression plasmid. The specific infectivities of the viruses were then determined, and the results are shown in Fig. S3 in the supplemental material. At the highest dose of mA3 plasmid tested, the specific infectivities of both viruses were decreased by a factor of 10, thus demonstrating the expected reduction in infectivity due to mA3. However, the slopes of the mA3 titration curves for the viruses with or without glycogag were similar. Thus, glycogag did not affect their sensitivity to mA3 under these experimental conditions.

### MLV lacking glycogag is blocked before or at reverse transcription.

The role of glycogag in infection would be much clearer if the specific defect leading to failure of infection in glycogag-negative virions could be identified. Pizzato reported (11) that a late step in reverse transcription was not completed upon infection by these particles. We have extended this finding by assaying for minus-strand strong-stop DNA, the initial product of reverse transcription. As shown by the results in Fig. S4 in the supplemental material, glycogag-negative particles (carrying xenotropic Env) with ~1/20 the specific infectivity of their glycogag-containing counterparts produced ~1/20 as much minus-strand strong-stop DNA as the glycogag-positive controls. As checks on the validity of these results, we also tested each virus preparation at a 1:5 dilution and after heat inactivation. As shown by the results in Fig. S4, in each case the diluted virus generated ~fivefold fewer DNA copies than the undiluted virus, while heat treatment reduced the copy numbers to the background level seen in mock-infected cultures. These controls show that copy numbers directly reflect the amount of infectious virus applied to the cultures. There was no significant contamination of the DNA preparations with DNA from the plasmids used to produce the viruses. We conclude that those particles that are noninfectious because they lack glycogag are unable to initiate DNA synthesis: infection is blocked at or before the beginning of reverse transcription.

### Measurement of MLV entry into susceptible cells.

In order to localize the block more precisely, we needed a way to monitor the entry of MLV particles into new host cells. One such assay has been described by Kolokotsov and Davey (32), but we found that its sensitivity was limited. (Many studies on HIV-1 entry have used the Vpr-BLaM assay [33, 34], but this requires Vpr or an equivalent protein, which is not available in MLV.) We have developed a new assay, similar to those reported by Voelkel et al., Kaczmarczyk et al., and Rosa et al. (25, 35, 36), in which the Cre recombinase enzyme is fused to MLV Gag protein and incorporated into virions; when these virions enter cells, the Cre in them catalyzes recombination in a reporter gene construct, leading to the expression of firefly luciferase. As detailed in Materials and Methods, the Cre-coding sequence was placed at the C terminus-coding end of the MLV Gag gene in the codon-optimized Gag expression vector, with an MLV protease cleavage site between the end of the NC gene and the Cre-coding sequence. (The Gag precursor is normally cleaved four residues before the C terminus of the protein [37], and thus, there are really two protease cleavage sites between the NC- and Cre-coding sequences in this construct.) The reporter was introduced into cells by either transient or stable transfection. The assay is depicted schematically in Fig. S5A in the supplemental material.

In an initial test of this assay, HT1080/mCAT1 cells were stably transfected with the reporter construct; we refer to these cells as “Cre reporter cells” below. They were then challenged with MLV particles produced with xenotropic Env or with no Env. As shown by the results in Fig. S5B in the supplemental material, the level of luciferase in the cells “infected” by virions lacking Env was similar to that in the mock-infected controls; in contrast, particles containing Env promoted luciferase expression by nearly 100-fold.

Further evidence that the increase in luciferase expression upon infection represents *bona fide* entry that uses the authentic cell surface receptor is shown in Fig S6A and B in the supplemental material. NIH3T3 mouse cells engineered to express the xenotropic MLV receptor, XPR1, as well as control NIH3T3 cells, which lack a receptor for xenotropic MLV, were transiently transfected with the reporter construct and then challenged with MLV particles containing the Gag-Cre fusion and either no Env, ecotropic Env, or xenotropic Env. As shown by the results in Fig. S6B, the luciferase level in the cells expressing XPR1 and infected with virus with xenotropic Env was significantly higher than the levels seen in the three controls, i.e., in cells without the XPR1 receptor (Fig. S6B, NIH/control) or in cells (with or without the receptor) infected with Cre-containing virus particles lacking Env. The values in these control samples were all similar to each other. In contrast, Cre-containing virus with an ecotropic Env gave similar luciferase values in cells with or without XPR1, as expected for these NIH3T3 cells, which naturally express the ecotropic receptor. Thus, both a functional Env protein and the presence of the appropriate receptor on the target cells are required for promotion of luciferase expression. As shown by the results in Fig. S6A, infectivity measurements on these virus preparations gave results completely consistent with these entry data.

Gammaretroviruses exhibit so-called “superinfection interference,” in which cells productively infected with a virus are strongly resistant to reinfection by a second virus targeted to the same receptor as the first; in fact, this phenomenon has been used to classify MLVs into groups with common receptor specificities (38, 39). Presumably, the receptor in the virus-producing cell is saturated by the Env protein synthesized within the cell, rendering it unavailable for a superinfecting virus particle. We also tested the ability of the new assay to detect this block to virus entry. Cre reporter cells were infected with the wild-type, replication-competent Moloney MLV (which encodes ecotropic Env) and passaged for 2 weeks, enabling the virus to spread throughout the culture. A control culture was mock infected and passaged in parallel with the infected culture. The cells were then challenged with virus containing the Gag-Cre fusion and bearing either no Env, ecotropic Env, or xenotropic Env. As shown by the results in Fig. S6D, virus with no Env induced no luciferase expression, while virus with xenotropic Env induced luciferase expression in both the preinfected and the control cultures. However, the virus with ecotropic Env induced luciferase in the uninfected control culture but not in the culture that was preinfected with ecotropic MLV. By demonstrating the entry block associated with superinfection interference, these results support the validity of the new entry assay. Again, the infectivity measurements (see Fig. S6C) were in full agreement with these entry results.

We also tested the assay by determining whether the expression of the luciferase from the Cre reporter requires an active reverse transcriptase. MLV containing the Gag-Cre fusion and a xenotropic Env but with a DD224-225AA mutation in the active site of reverse transcriptase (40, 41) was generated and used to infect the Cre reporter cells. Although this virus was devoid of infectivity, as expected (see Fig. S6E in the supplemental material), it gave a positive result in the entry assay (see Fig. S6F). Thus, luciferase expression from the reporter is independent of reverse transcription, lending further support to its validity as an entry assay. This experiment also included a control that demonstrated that the “entry” signal measured here requires the inclusion of Gag-Cre during virus production, as expected.

### Effects of glycogag and S2 upon virus entry.

**FIG 7:**
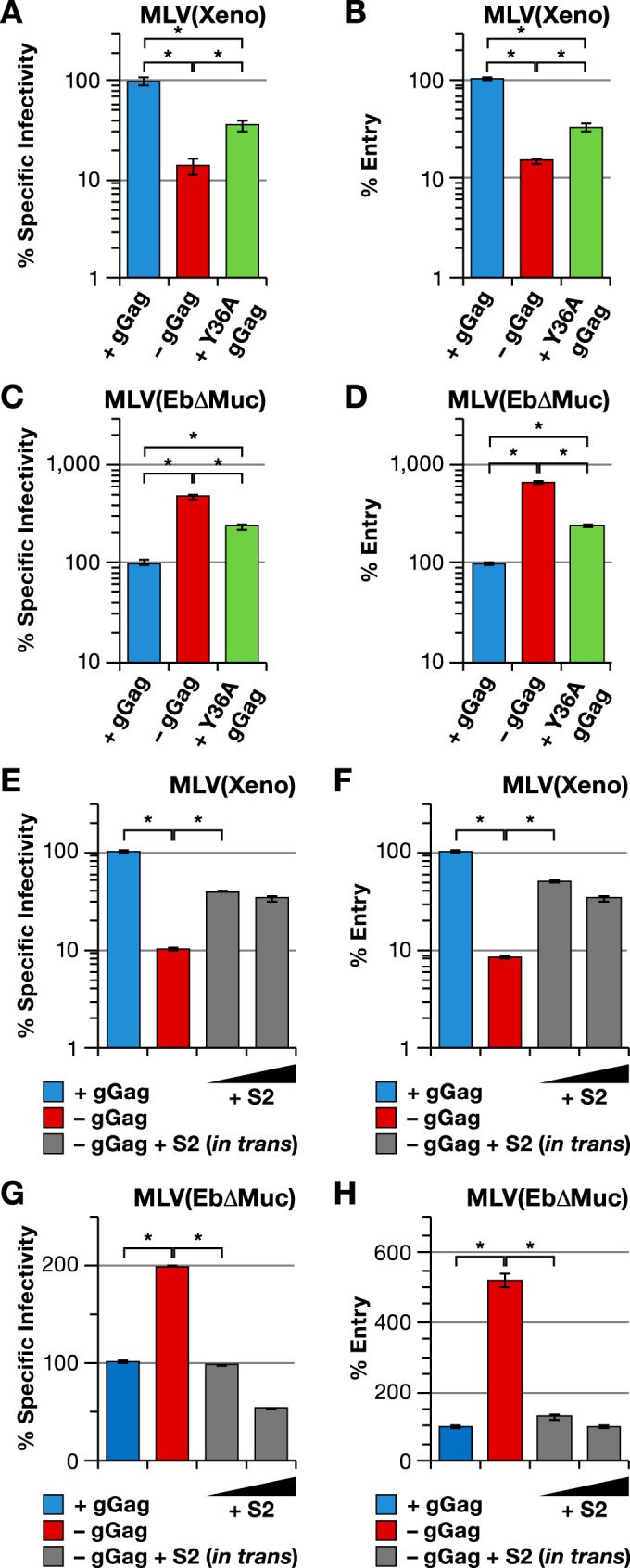
Glycogag and EIAV S2 modulate MLV entry. Specific infectivity (left) and entry (right) of MLV with wild-type Gag-Pol (blue bars), mutant Gag-Pol lacking gGag (red bars), mutant Gag-Pol with Y36A mutation in gGag (green bars), and mutant Gag-Pol lacking gGag produced in the presence of S2 expression plasmid (grey bars). The viruses were produced with Xeno (xenotropic) envelope (A, B, E, and F) or EbΔMuc (C, D, G, and H) and assayed on the Cre reporter cell line. The S2/Gag-Pol plasmid ratios used in the experiments whose results are shown in panels E to H were 1:27 and 1:9. *, *P* < 0.0001.

We then used this assay to determine whether virions that were deficient in infectivity because they lacked glycogag were capable of entering new host cells. Viruses carrying a xenotropic Env either with or without glycogag were produced in the presence of the Gag-Cre fusion protein. As the entry assay uses firefly luciferase, infectivities in this experiment were measured using a NanoLuc luciferase reporter vector, rather than the firefly luciferase vector used in the majority of our experiments. As shown by the results in Fig. 7A, the glycogag-containing virus had a specific infectivity (as measured on HT1080 cells) that was significantly higher than that of the virus lacking glycogag, as expected. Figure 7B shows the results of the entry assay with these two virus preparations, as assayed on HT1080 cells containing the Cre-dependent luciferase reporter. It can be seen that the entry capability of the glycogag-containing virus was correspondingly higher than that of the virus lacking glycogag. Therefore, virions which cannot infect cells because they lack glycogag and carry a xenotropic Env are blocked at entry into new host cells.

As shown by the results in Fig. 4, glycogag is deleterious for MLV with Ebolavirus GP (with or without a deletion of its mucinlike domain). We also tested the possibility that this deleterious effect was exerted at the level of entry into the host cell. As shown by the results in Fig. 7C and D, virus with the Ebolavirus GP lacking its mucinlike domain (EbΔMuc) and with glycogag is deficient in entry to roughly the same extent as it is deficient in specific infectivity; thus, while glycogag enhances the entry of MLV particles with xenotropic Env, it impairs the entry of particles with the Ebolavirus GP.

As described above, the replacement of Y36 of glycogag with alanine diminishes but does not completely eliminate its ability to enhance the infectivity of MLV(Xeno) (Fig. 6). We produced virions using a Gag-Pol plasmid in which the tyrosine codon in the glycogag-coding region had been mutated to an alanine codon. As shown by the results in Fig. 7A, this virus was intermediate in its specific infectivity between virus with wild-type glycogag and virus with no glycogag, as expected. This difference was also evident in the ability of the virions to enter new host cells (Fig. 7B). Similarly, MLV(Ebola) produced with Gag-Pol containing the Y36A mutation was intermediate between virus with no glycogag and that with wild-type glycogag, with respect to both specific infectivity (Fig. 7C) and entry (Fig. 7D).

We also tested the effect of EIAV S2 expression in these experiments. As shown by the results in Fig. 7F, S2 enhances the entry capability of MLV(Xeno), consistent with its increased specific infectivity (Fig. 7E). Conversely, the presence of EIAV S2 in cells producing MLV(Ebola) reduces the entry capability (Fig. 7H), as well as the infectivity (Fig. 7G), of the virus (as ΔMuc Ebola glycoprotein was used in this experiment, the effects seen here were somewhat smaller than those seen with full-length glycoprotein). Taken together, the results show that glycogag and S2 exert their effects upon the specific infectivity of MLV(Xeno) and MLV(Ebola) largely, if not entirely, by modulating the ability of the virions to successfully enter new target cells.

### Effects of ectopic expression of Serinc5 upon viral infectivities and virus entry.

**FIG 8:**
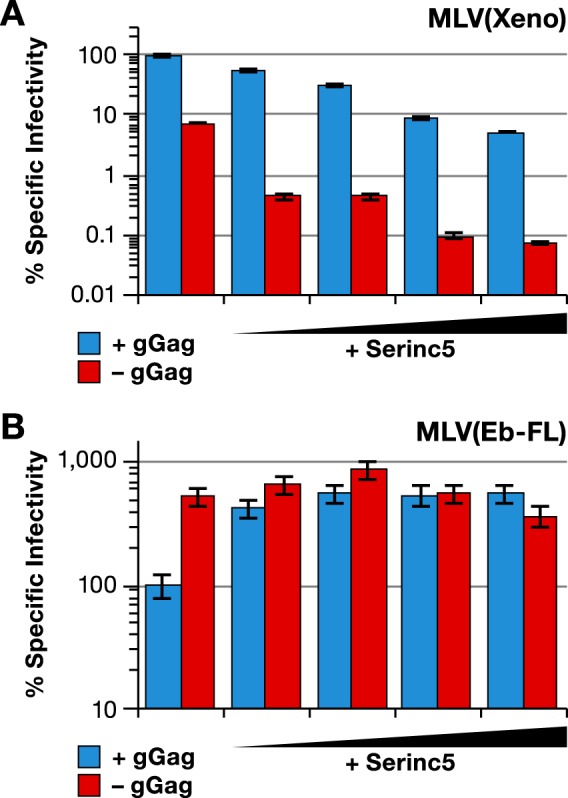
Antagonism between Serinc5 and glycogag with respect to MLV(Xeno) and MLV(Ebola) infectivity. Specific infectivities of MLV with wild-type Gag-Pol (blue bars) or mutant Gag-Pol lacking gGag (red bars) produced in the absence or presence of increasing amounts of Serinc5 expression plasmid and with Xeno (A) or Eb-FL (B) glycoprotein are shown. The Serinc5/Gag-Pol plasmid ratios used were increased by threefold increments from 1:81 to 1:3. The infectivity measurements were performed on the HT1080/mCAT1 cell line.

As glycogag has been shown to antagonize Serinc5 (25, 26), it was of interest to determine the effect of Serinc5 expression upon the infectivities of MLV(Xeno) and MLV(Ebola) virions. Viruses were produced by transfection of 293T cells with graded doses of a Serinc5 expression plasmid, in addition to the plasmids encoding the viral constituents and pBabeLuc. As shown by the results in Fig. 8A, the stepwise addition of Serinc5 plasmid drastically reduced the specific infectivity of MLV(Xeno) in the absence of glycogag (Fig. 8A, red bars), as previously reported by Rosa et al. (25). At the lowest Serinc5 dose tested, the specific infectivity of the virus without glycogag decreased by ~20-fold, whereas that of the virus with glycogag (Fig. 8A, blue bars) decreased by less than twofold. The highest dose of Serinc5 resulted in an ~100-fold loss of specific infectivity of glycogag-negative virus relative to the amount of virus produced with no added Serinc5. In contrast, glycogag-positive virus, whose initial specific infectivity was ~10-fold higher than that of the virus without glycogag, only suffered a ~20-fold loss of specific infectivity at the highest Serinc5 dose. Thus, glycogag mitigates the adverse effects of Serinc5 expression upon MLV(Xeno) infectivity.

We also performed an analogous Serinc5 titration in cells producing MLV(Ebola) with and without glycogag. As shown by the results in Fig. 8B, the lowest dose of Serinc5 significantly enhanced the specific infectivity of MLV(Ebola) containing glycogag (Fig. 8B, blue bars), and higher levels of Serinc5 had no additional effect. In contrast, we saw no effect of Serinc5 upon glycogag-negative virus (Fig. 8B, red bars).

**FIG 9:**
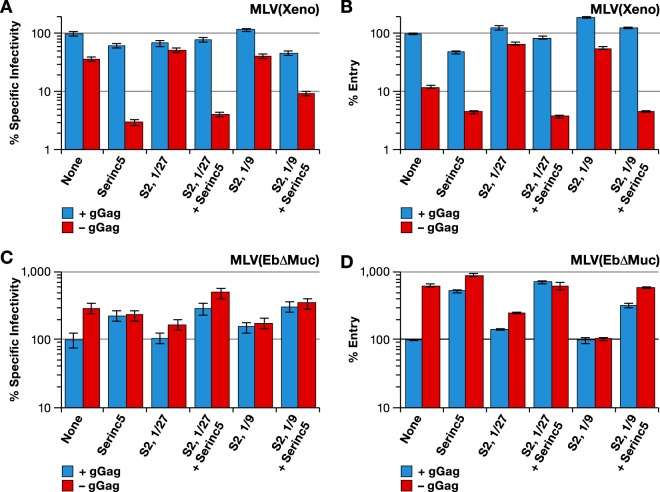
Serinc5 modulates the entry of MLV(Xeno) and MLV(Ebola). Specific infectivities (left) and entry (right) of MLV with wild-type Gag-Pol (blue bars) or mutant Gag-Pol lacking gGag (red bars) produced with Xeno (A and B) or EbΔmuc (C and D) glycoprotein together with control plasmid (none) or Serinc5 expression plasmid (Serinc5), with or without S2 expression plasmid used at a 1:27 or 1:9 ratio to Gag-Pol plasmid. The viruses for specific infectivity and entry measurements were produced in parallel transfections. Specific infectivity and entry measurements were performed on HT1080/mCAT1 cells and Cre reporter cells, respectively.

The effect of Serinc5 expression upon virus entry was also tested. MLV with xenotropic Env was produced, with or without glycogag or EIAV S2 and with or without Serinc5. As shown by the results in Fig. 9A, the expression of Serinc5 in the absence of glycogag or S2 resulted in an ~10-fold reduction in specific infectivity, as expected from the results shown in Fig. 8. This was accompanied by a similar drop in entry into the target cells (Fig. 9B). In contrast, with glycogag-positive virus (Fig. 9B, blue bars), both specific infectivity and entry were unaffected by Serinc5. We did not detect an effect of S2, cotransfected with Gag-Pol, upon the entry or infectivity of the virus lacking gGag in the presence of Serinc5. A similar experiment was performed to test the effect of Serinc5 on MLV(Ebola) produced with or without glycogag or EIAV S2. Low titers in the entry assay with MLV carrying full-length Ebola glycoprotein necessitated the use of the ΔMuc Ebola glycoprotein for this experiment. As shown by the results in Fig. 9C, the expression of Serinc5 enhanced the specific infectivity of MLV(EbΔMuc) produced in the presence of glycogag (Fig. 9C, blue bars) but had no effect on that of the virus produced without glycogag (Fig. 9C, red bars). Entry into the target cells of the virus produced in the presence of glycogag was similarly enhanced by Serinc5 (Fig. 9D). The results in Fig 9C and D also show that the expression of S2 during virus production decreased the specific infectivity and entry of glycogag-negative MLV (Ebola) into the target cells, an effect that was completely reversed in the presence of Serinc5. Expressing S2 at a threefold-higher dose (S2/Gag-Pol plasmid ratio of 1:9, rather than 1:27) did not interfere with the positive effect of Serinc5 on entry or specific infectivity.

## DISCUSSION

Although there are numerous proposals in the literature, the function(s) of MLV glycogag have remained elusive for many years. One factor complicating the genetic analysis of glycogag function has been the fact that its coding region within the MLV genome largely overlaps the viral *gag* gene. In the present work, we have designed vectors enabling the independent expression and detection of glycogag and Gag (Fig. 1). Using these tools, we documented the transit of glycogag from the rough ER to the Golgi apparatus, prior to its export to the cell surface (Fig. 2).

The most striking results presented here can be briefly summarized as follows. As reported by Pizzato and Usami et al., the presence of glycogag in virus-producing cells significantly affects the specific infectivity of gammaretroviral particles under certain conditions (11, 24). We attempted to define these conditions as precisely as possible. We found (in agreement with Pizzato [11]) that one major factor determining the glycogag requirement was the Env protein used by the virus (Fig. 4). Thus, glycogag had little or no effect on the specific infectivity of MLV particles with ecotropic MLV Env, RSV subgroup A Env, BLV Env, or the surface glycoprotein of VSV. In contrast, it significantly increased the specific infectivity of MLV particles if the surface glycoprotein was from amphotropic (11), xenotropic (11), or 10A1 MLV, the gammaretroviruses RD114 and GALV, or HIV-1 (11). Finally, it reduced the specific infectivity of MLV particles carrying Ebolavirus glycoprotein. In those cases where it could be tested, the crucial variable determining whether glycogag was required was not the identity of the target cells but, rather, that of the Env and/or that of the receptor used by the Env for infection (Fig. 3). We also found (Fig. 5B and C) that EIAV S2 protein has the same effects as glycogag, although it appears to be somewhat less potent.

We devised a new assay to measure the entry of MLV into the cytoplasm of the cell (see Fig. S5 and S6 in the supplemental material). The results obtained with this assay showed that the effects of glycogag upon specific infectivity reflected the abilities of the different viruses to enter the cells. Experiments with a Serinc5 expression plasmid also showed that, as indicated by the studies of Rosa et al. and Usami et al. (25, 26), glycogag and Serinc5 are mutually antagonistic (Fig. 8). Therefore, the ability of a virus particle to penetrate into the cytoplasm of a target cell is strongly affected by the specific receptor targeted by the virus and by the uninhibited presence of Serinc5 in the virus-producing cell. More specifically, MLV with xenotropic Env protein (and presumably several other gammaretrovirus Env proteins) is impaired with respect to entry if it is produced in a cell expressing Serinc5 with no glycogag or EIAV S2; conversely, MLV with Ebola glycoprotein enters cells more efficiently if it is made under these conditions (Fig. 9). The fact that Serinc5 expression had no effect upon MLV(Ebola) in the absence of glycogag (Fig. 8) strongly suggests that the antagonism between Serinc5 and glycogag is sufficient to explain the effects of the latter. In turn, this implies that all or nearly all of the effects of glycogag observed in the absence of added Serinc5 reflect its interactions with the Serinc5 naturally expressed in the 293T cells we used for virus production. We also cannot exclude the possibility that other factors, including other Serinc family members (27), contribute to these effects.

Using a similar assay, Rosa et al. (25) reported that Nef-deficient HIV-1 was impaired in entry into the cell but that this block was smaller in magnitude than the block to infectivity. They suggested that some infecting virions create fusion pores large enough to permit passage of Cre but too small for a viral core to penetrate. We frequently see a similar discrepancy between our entry and infectivity results (e.g., Fig. 9).

We noted that remarkably low levels of the glycogag expression plasmid, ≤1% of the amount of the Gag-Pol plasmid, were sufficient to restore nearly full infectivity to MLV carrying xenotropic Env protein (Fig. 5A). This suggests that a relatively small amount of glycogag protein is sufficient to counteract the endogenous Serinc5 in 293T cells. It seems likely that in the natural context, initiation of glycogag translation from the noncanonical CUG initiation codon is very inefficient, yielding a very low ratio of glycogag-to-Gag synthesis. Remarkably, the enhancement by glycogag of the infectivity of MLV(Xeno) or of Nef-deficient HIV-1 seems to require only the N-terminal 66 amino acids, which are in the portion of glycogag not shared with Gag (24). Thus, rather than being “another form of Gag,” glycogag appears to represent a true accessory protein of MLV: the Gag sequences in it are not necessary for its functions.

We do not know the mechanism of the functional interaction between Serinc5 and the viral entry machinery. While little is known about Serinc5, its properties suggest that it might influence phospholipid metabolism (27) and, thus, affect the lipid composition of released virions. In fact, it has been reported that glycogag targets MLV assembly toward lipid rafts on the surface of virus-producing cells and, thus, alters the lipid profile of MLV particles (8). It seems possible that the lipid composition of the virus could influence the efficiency of virus entry for a given envelope-receptor pair. Alternatively, perhaps Env, in concert with glycogag, partially determines the site of viral budding and, hence, the lipid composition of the virus; this effect of Env might be analogous to what is seen in polarized epithelial cells, in which the assembly site of a retrovirus is dictated by Env (42, 43). While we did not detect an effect of glycogag upon the lipids responsible for annexin V binding (see Fig. S2 in the supplemental material), it is still possible that it affects other aspects of the lipid profile of the virions. We also noted that the effects of glycogag upon the infectivity of and cell entry by MLV(Xeno) and MLV(Ebola) were reduced if the tyrosine at position 36 was replaced by alanine (Fig. 6 and 7). As this tyrosine is in a YXXL motif, which might function in endocytosis (24), these results are consistent with the proposal (25, 26) that the mechanism of these glycogag actions involves targeting Serinc family proteins to an endosome.

We also do not know why the contribution of glycogag to infectivity is so large for viruses with some Env proteins but insignificant for those with other Env proteins. Most of those in the former group are believed to induce direct fusion of the viral membrane with the plasma membrane of the new host cell, while the latter group includes proteins such as VSV(g), which cause membrane fusion only in the acidic environment of the endosome. The latter group also includes ecotropic MLV Env, whose fusion mechanism is somewhat controversial: it has been reported to show sensitivity to lysosomotropic agents, like pH-sensitive viruses (44–46), but other data argue strongly against a pH-sensitive mechanism in ecotropic MLV entry (32, 47, 48). RSV entry also entails a pH-sensitive step (47).

The interactions of MLV carrying Ebolavirus glycoprotein with glycogag and Serinc5 were unique in our experiments. It is interesting that a principal attachment factor for these virions is the cell surface protein TIM-1 (49) and that this protein binds PS (50). It seemed possible that, through its effects on Serinc family proteins and, thus, on phospholipid metabolism, glycogag reduces PS levels on virion surfaces. This might reduce the efficiency of interactions between the virions and TIM-1 on target cells. However, as just mentioned, we saw no effect of glycogag upon annexin V binding to virions. It is still conceivable that glycogag affects PS levels but that annexin V binding to PE obscures this (29). It is important to note that Ebolavirus penetrates cells by a complex, circuitous route, very different from that of any known retrovirus (51–53). This entry pathway includes cleavage of the Ebolavirus glycoprotein by a cathepsin. We tested MLV with either full-length Ebolavirus glycoprotein or the glycoprotein lacking its mucinlike domain; this form gives rise to significantly higher titers of MLV pseudotypes (21, 22). Interestingly, particles with EbΔMuc glycoprotein tended to have smaller responses to glycogag and Serinc5 than those with full-length Ebolavirus glycoprotein (e.g., Fig. 4 and 9). We did not detect an effect of EIAV S2 protein upon infection and entry by MLV(Ebola) particles in the presence of Serinc5 plasmid (Fig. 9C and D); this negative result might reflect a qualitative difference between the activities of S2 and those of glycogag or might merely result from a lower level of activity for S2 than for glycogag.

Some of the data presented here appear to conflict with other published reports [e.g., the negative mA3 results (see Fig. S3 in the supplemental material)]. It seems likely that these discrepancies reflect differences in how and where the viruses were generated. We produced virus by transient expression of viral components in 293T cells, while in other studies, virus was generated in mice or in infected mouse cells (6, 7, 30). Transient expression in non-mouse cells prevents contributions from or interactions with other endogenous MLV genomes or constituents; the source of the virus could obviously affect its lipid composition as well.

It is striking that glycogag complements a Nef defect in HIV-1, although the two proteins show no obvious relationship. (However, the converse is apparently not true: Nef has not been observed to restore the infectivity of glycogag-deficient MLV [11]). There is also almost no similarity between glycogag and EIAV S2. The fact that MLV, HIV-1, and EIAV have independently evolved proteins that counteract Serinc5 underscores the importance of this function for retroviral infectivity. It will be important to learn more about the effects of these proteins on both retroviral and filoviral functions. It seems possible that comparative studies on these viruses and on the nonreciprocal complementation between glycogag and Nef will be particularly instructive in this regard.

## MATERIALS AND METHODS

### Plasmids.

Two plasmids were made by modifying our full-length, infectious proviral clone of Moloney MLV (54), which was originally obtained from the late Richard J. Mural. In one, the *env* gene was inactivated by filling in the BstEII site at nucleotide (nt) 5223; this 5-base insertion introduced a stop codon early in the Env-coding region (55). All of the “Gag-Pol” plasmids used in this study were this full-length proviral clone with this mutation destroying the Env open reading frame (ORF). In the other modification, the glycogag-coding region was inactivated: we introduced stop codons at nt 450 and 519, in frame with the glycogag and Gag genes; these termination codons interrupt translation from the glycogag initiation codon at nt 357 but do not affect the synthesis of Gag or other viral proteins.

A plasmid expressing only glycogag [designated pCMV(glycogag)] was constructed as follows. We obtained a plasmid expressing, in pcDNA3.1(+), a codon-optimized *gag* gene from xenotropic murine leukemia virus-related virus (XMRV); this plasmid was a kind gift from Hanni Bartels and Jeremy Luban. We first introduced the sequences encoding the glycogag-specific portion of Moloney MLV glycogag by adding nt 357 to 620 from Moloney MLV. We further modified the resulting plasmid by replacing the C residue at nt 357 with A and by replacing the AUG at nt 621 to 623 with GCC. In addition, nucleotides encoding a *myc* epitope tag (GEQKLISEEDLG) were introduced in this clone between codons 45 and 46 of the p12-coding region; this site is tolerant to insertions in MLV (12–14). The experiments described in this report use this plasmid, which encodes the chimeric glycogag in which residues 1 to 88 are from Moloney MLV, while the remaining 538 residues are from XMRV; however, many of them have been repeated with a plasmid encoding glycogag derived entirely from Moloney MLV. No differences in the properties of these two glycogags have been detected.

We also inserted a FLAG epitope tag (YKDDDDK) in place of residues 46 to 52 of the p12-coding region in the XMRV Gag expression plasmid. The plasmid mCh-Sec61 beta, an expression plasmid for the ER marker Sec61 beta, was a gift from Gia Voeltz (plasmid 49155; Addgene). In all cases, the sequence of the coding regions in each plasmid was confirmed.

The following plasmids have all been described previously: pCD-Env, expressing Moloney MLV Env; pBabe-Luc, an MLV vector expressing firefly luciferase; the MLV vector expressing green fluorescent protein (GFP); and the mA3 expression plasmid (56–59). The plasmid pLXSH-nLucP, an MLV vector expressing nanoLuc-PEST, was constructed by amplifying the nanoLuc-PEST-coding region from pNL-1.2 NLucP (Promega) and cloning it into the pLXSH vector (60) between the HpaI and BamHI restriction sites. We also used expression plasmids for the following: xenotropic MLV Env (a kind gift of Heinrich Göttlinger) (24); RD114 Env (pCI-RD114) (61) (a kind gift from Manuel Caruso); gibbon ape leukemia virus (GALV) (pCIneo-GALV-SEATO) Env (59) (a kind gift from Maribeth Eiden); Ebolavirus glycoprotein (pCAGGS EboGPz) (62) and Rous sarcoma virus subgroup A Env (pCB6-EnvA) (63), both kind gifts of Paul Bates; BLV Env (a kind gift of Jean-Luc Battini and Marc Sitbon); Ebolavirus GP with a deletion of its mucinlike domain (a kind gift of Judith White) (21, 22); the xenotropic MLV receptor from human cells (pLNC3XflagXPR1) (64–66) (a kind gift of Maribeth Eiden); and the ecotropic MLV receptor from mouse cells (pcDNA3.1-mCAT1) (67) (a kind gift of Lorraine Albritton). A pcDNA3-based plasmid expressing EIAV S2 protein was a kind gift of Fred Fuller. The Cre reporter plasmid (a kind gift from Stan Kaczmarczyk) was p231 (68), in which noncoding sequences from pBS302 (69), flanked by LoxP sites, are followed by the firefly luciferase gene. The plasmid encoding an MLV Gag-Cre fusion was constructed by amplifying the Cre-coding region, together with a nuclear localization signal, from pML78 (70) (a kind gift of Mark Lewandoski) and cloning it into the XMRV Gag expression vector. Sequences coding for an MLV protease cleavage site (TSQAFPLRAG) were placed between the last codon of the *gag* gene and the Cre-coding region. The Serinc5 expression plasmid, in the vector pBJ5, was a kind gift of Heinrich Göttlinger (26). The component plasmids of the *piggyBac* transposon-based expression system (17), including pCyL43, pB-RB, and pB-T-Rfa, were a kind gift from Andras Nagy.

### Cells and viruses.

Cell lines were maintained in Dulbecco modified Eagle medium (DMEM) supplemented with 10% fetal calf serum and penicillin-streptomycin. NIH3T3/hXPRI mouse cells were created by stable transfection with pLNC3XflagXPR1 and selection of G418-resistant cells. HT1080 cells were a kind gift from Heinrich Göttlinger. mCAT1 was introduced into these cells by stable transfection with pcDNA3.1-mCAT1 and selection of G418-resistant cells. In order to construct Cre reporter cells, the Cre reporter p231 was introduced into HT1080/mCAT1 cells by stable cotransfection with the selectable plasmid pcDNA*Zeo*, followed by selection of zeocin-resistant cells. CHO/hXPRI hamster cells were a kind gift from Marc Sitbon. RSV subgroup A Env was assayed on D17-tva dog cells, a kind gift from Stephen Hughes.

In some experiments, glycogag was expressed in a stable cell line under doxycycline control. This was done by moving the glycogag-coding region (with *myc* tag) from pCMV(glycogag) into the *piggyBac* transposon-derived plasmid PB-T-Rfa (17) and introducing it into HeLa cells as described previously (17). Control cells containing the empty transposon vector were selected in parallel.

In all experiments, viruses were produced by transient transfection of 293T cells using Trans-IT 293 transfection reagent (Mirus Bio LLC) according to the manufacturer’s recommendations. Unless noted otherwise, the cells were cotransfected with a mixture of an Env-defective MLV clone (producing Gag-Pol, with or without an intact glycogag gene), pBabeLuc, and an Env expression plasmid. All supernatants were collected at 48 and 72 h posttransfection, pooled, and filtered through 0.45-µm filters.

Except where specified otherwise, infectivity was assayed by measurement of firefly luciferase activity in cell lysates 48 h after infection, as described in Rulli et al. (57). Specific infectivity was determined by normalizing the infectivity to the quantity of virus, measured by immunoblotting for p30^CA^ directly on the filtered culture fluids as described below. The effects of mA3 on infectivity were determined as described previously (57).

### Determination of PS on virion surfaces.

Virions were prepared and assayed exactly as described previously (28).

### Immunoblotting.

The antisera used included rabbit anti-p30^CA^, rabbit anti-*c*-myc (C3956; Sigma), and mouse anti-FLAG (F3165; Sigma) antisera as primary antibodies, followed by appropriate antibody-horseradish peroxidase (HRP) conjugates as secondary antibodies for chemiluminescent detection. Quantitation of virus in cell culture supernatants was done by near-infrared quantitative Western blot analysis, utilizing reagents from LI-COR Biosciences according to the manufacturer’s instructions. After separation on SDS-PAGE gels, proteins were transferred to low-background Immobilon-FL transfer membranes (Millipore). The membranes were incubated overnight at room temperature with rabbit anti-p30^CA^ antiserum. IRDye 680RD donkey anti-rabbit antiserum (925-68073; LI-COR Biosciences) was used as the secondary antibody. Membranes were imaged with the Odyssey Imaging system to detect p30 bands, followed by quantitation of the amount of p30 using Image Studio Lite version 4.0 (LI-COR Biosciences). In each experiment, the signal was shown to be in the linear range by comparison with dilutions of a known virus-containing sample on the same gel.

### Immunofluorescence and confocal microscopy.

The antibodies used for immune staining were as follows: rabbit anti-myc antibody (C3956; Sigma); mouse anti-FLAG antibody (F3165; Sigma); and rabbit monoclonal anti-GM130 antibody (ab52649; Abcam, Inc.). Primary antibodies were detected with goat anti-rabbit-488A (20019; Biotium) and donkey anti-mouse-594 (20115; Biotium) antibodies. Amounts of 2.5 × 10^4^ to 5 × 10^4^ cells were seeded in 35-mm poly-l-lysine-coated cell culture dishes (P35GC-0-14-C; MatTek) the day before transfection or the addition of doxycycline. After 24 h of doxycycline treatment or 24 h after transfection, cells were rinsed once with phosphate-buffered saline (PBS), fixed with fixation buffer (22015; Biotium) for 10 min at room temperature (RT), rinsed once with PBS, incubated with 50 mM NH_4_Cl at RT for 5 min, and then incubated with 0.2% Triton X-100 in PBS at RT for 5 min. Cells were rinsed once with PBS containing 1% bovine serum albumin (PBS-BSA) and incubated in PBS-BSA at RT for 15 min. Primary and secondary antibodies were diluted in PBS-BSA. Primary antibodies were incubated for 1 h at 37°C, followed by three washes with PBS-BSA. Secondary antibodies were incubated at RT for 1 h, followed by three washes with PBS-BSA. Cells were mounted with Prolong anti-fade mounting reagent with DAPI (4′,6-diamidino-2-phenylindole) (Invitrogen). Confocal microscopy was performed using a Zeiss LCI510 confocal microscope, and images were analyzed using the LSM Image Browser (Zeiss).

### Measurement of viral DNA synthesis.

The synthesis of minus-strand strong-stop MLV DNA was assayed as follows. HT1080 cells were seeded in 6-cm cell culture dishes at 2.5 × 10^5^ cells/dish. Cells were infected 24 h later. Before infection, the amounts of virus in each sample were equalized by quantitative anti-p30 antibody immunoblotting. To eliminate plasmid DNA carryover from transfection, the virus-containing cell culture medium was incubated with 20-U/ml DNase I (New England Biolabs) in the presence of 4 mM MgCl_2_ for 1 h at 37°C. An aliquot of DNase I-treated virus was treated at 68°C for 20 min and used in a control infection. Twenty-four hours after infection, total DNA was extracted from infected cells using the QIAamp DNA blood minikit (Qiagen) following the manufacturer’s instructions. The extracted DNA was further treated with DpnI (New England Biolabs) to digest any remaining plasmid DNA carried over from transfection. The numbers of strong-stop DNA copies in the extracted DNA were determined by real-time PCR. The final concentrations of the reagents in the PCRs were 1× PCR buffer II (Invitrogen), 4 mM MgCl_2_, 200 µM deoxynucleoside triphosphates (dNTPs), 600 nM MLV-SSF4 (5′ CCGTGTATCCAATAAACCCTCTT 3′), 600 nM MLV-SSR2 (5′ TAGTCAATCACTCAGAGGAGACC 3′), 50 nM P-SSMLV-1 probe (5′-FAM-ATCCGACTTGTGGTCTCGCTGTTCCT-TAMRA-3′ [FAM, 6-carboxyfluorescein; TAMRA, 6-carboxytetramethylrhodamine]), and 0.625 U AmpliTaq Gold polymerase (Invitrogen) in a 25-µl volume. The reaction mixtures were heated to 95°C for 10 min, followed by 35 cycles of 95°C for 30 s and 56°C for 30 s.

### Virus entry assay.

Viruses for the entry assay were produced by transient transfections of 293T cells with the following plasmids: gGag^+^ or gGag^−^ MLV Gag-Pol plasmid, an MLV-based vector, an Env expression plasmid, and the Gag-Cre fusion plasmid. The ratio of Gag-Pol plasmid to Gag-Cre fusion plasmid was 5:1. Supernatants were collected at 48, 72, and 96 h posttransfection, pooled, and filtered through 0.45-µm filters. Virus entry was measured by infecting cells containing the Cre reporter and assaying the infected cells for firefly luciferase activity 48 h later. The luciferase signals were normalized to the quantity of virus to calculate the relative rates of entry. Because firefly luciferase was used as the reporter for entry, an MLV-GFP vector (56) or pLXSH-nLucP was often used in place of firefly luciferase for specific infectivity measurements. When the MLV-GFP vector was used, the number of GFP-positive cells was determined with a fluorescence-activated cell sorter 48 h postinfection and normalized to the quantity of virus. When pLXSH-nLucP was used, the infectivity measurements were performed using the Nano-Glo luciferase assay (Promega) according to the manufacturer’s instructions, and the Nano-Glo luciferase signal was normalized to the quantity of virus. In other experiments, pBabeLuc was used as the reporter for infectivity. Since in these cases both the infectivity and entry measurements used firefly luciferase as the readout, they were performed on parallel samples rather than identical samples as in the GFP or nLuc assays.

### Statistics.

Specific infectivity and entry measurements are shown as the arithmetic means ± standard deviations (SD) of luciferase signals measured in triplicate after normalization to the amount of virus in the samples. Unpaired *t* tests were performed to determine significance and obtain *P* values for these results. In many cases, brackets indicating *P* values were omitted from the figures for clarity. Each experiment was performed independently at least twice, and the results of a representative experiment are shown. Unless otherwise specified, the specific infectivity and entry of the virus produced with glycogag is set to 100.

## SUPPLEMENTAL MATERIAL

Figure S1  Glycogag does not colocalize with Gag. HeLa/vector or HeLa/gGag cells were transfected with mock or Gag-FLAG expression plasmid. At 24 h posttransfection, gGag expression was induced by adding doxycycline at 10 ng/ml and continued for the next 24 h. The cells were then stained with anti-Myc and anti-FLAG antibodies for detection of gGag and Gag, followed by confocal microscopy. DAPI was used for staining nuclei. Download Figure S1, PDF file, 0.9 MB

Figure S2  Glycogag does not affect annexin V binding to virions. Relative PS levels on MLV(Eco) and MLV(Xeno) (A) and MLV(Eb-FL) (B) produced using wild-type Gag-Pol (blue bars) or mutant Gag-Pol lacking gGag (red bars). The units of the relative values are arbitrary, and values cannot be compared between panels A and B. Download Figure S2, PDF file, 0.01 MB

Figure S3  Glycogag does not affect sensitivity of MLV(Xeno) to mA3. Specific infectivity of MLV(Xeno) produced with wild-type Gag-Pol (blue line) or with the mutant Gag-Pol lacking gGag (red line) by transient transfection of 293T cells in the presence of increasing amounts of mA3 expression plasmid. Download Figure S3, PDF file, 0.02 MB

Figure S4  Viral DNA synthesis by MLV(Xeno) with or without glycogag. (A) MLV was produced by transfection of 293T cells with wild-type Gag-Pol (blue bars) or the mutant Gag-Pol lacking glycogag (red bars) together with the xenotropic Env expression plasmid. HT1080 cells were then infected with these viruses, either undiluted or diluted (1/5) or heat treatment inactivated (HT), or were mock infected. The cells were assayed 24 h later for MLV minus-strand strong-stop DNA. (B) Specific infectivities of viruses used in the experiment whose results are shown in panel A *, *P* < 0.0001. Download Figure S4, PDF file, 0.03 MB

Figure S5  Strategy of MLV entry assay. (A) Schematic depiction of the entry assay. The inset shows the expected cleavage pattern of the Gag-Cre protein. (B) Signal in the luciferase assay performed on Cre reporter cells at 48 h postinfection. The cells were mock infected or infected with MLV carrying Cre recombinase, either with no Env (red bar) or with Xeno Env (orange bar). Counts obtained in the luciferase assay measured in triplicate ± standard deviation are shown. Download Figure S5, PDF file, 0.02 MB

Figure S6  Validation of MLV entry assay. (A and B) Specific infectivities (A) and entry (B) of MLV with wild-type Gag-Pol, produced with no envelope (No Env) or Eco or Xeno envelope, assayed on NIH/control or NIH/XPR1 cells which had been transiently transfected with the Cre reporter cassette. (C and D) Cre reporter cells were pre-infected with MoMLV or were mock-infected and passaged for 2 weeks. The viruses used in the experiments whose results are shown in panels A and B were then assayed for specific infectivity (C) and entry (D) on these cells. In the specific infectivity graphs, the bars represent the percentage of GFP-positive cells normalized to the amount of virus in the samples. The entry and specific infectivity values are the percentages of the values for MLV(Eco). (E and F) Specific infectivity (E) and entry (F) of MLV with wild-type (blue bar) or DD224-225AA RT mutant (grey bar) Gag-Pol carrying Cre recombinase and Xeno Env, assayed on the Cre reporter cells. No Env, MLV with wild-type Gag-Pol but no Env glycoprotein; No Cre, MLV with wild-type Gag-Pol and Xeno Env but without Cre recombinase. *, *P* < 0.0001. Download Figure S6, PDF file, 0.02 MB
