# Supplementary figures and images for: Functional Interplay Between Murine Leukemia Virus Glycogag, Serinc5, and Surface Glycoprotein Governs Virus Entry, with Opposite Effects on Gammaretroviral and Ebolavirus Glycoproteins
Source: mBio. 2016 Nov 22;7(6):e01985-16. doi: 10.1128/mBio.01985-16 (PMC5120145; doi:10.1128/mBio.01985-16)

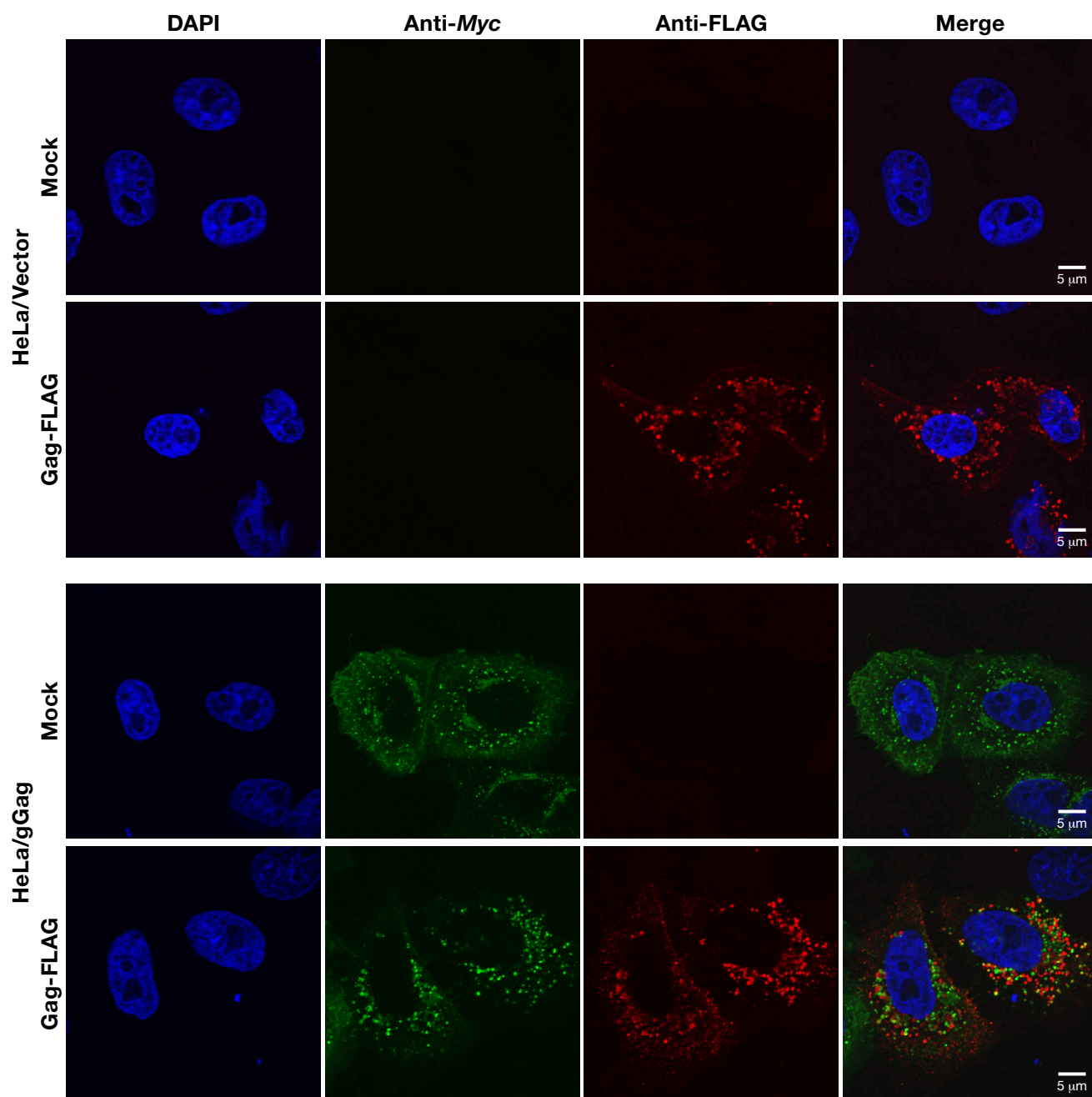

Supplement: Figure S1 — Glycogag does not colocalize with Gag. HeLa/vector or HeLa/gGag cells were transfected with mock or Gag-FLAG expression plasmid. At 24 h posttransfection, gGag expression was induced by adding doxycycline at 10 ng/ml and continued for the next 24 h. The cells were then stained with anti-Myc and anti-FLAG antibodies for detection of gGag and Gag, followed by confocal microscopy. DAPI was used for staining nuclei. Download [file mbo006163085sf1.pdf]

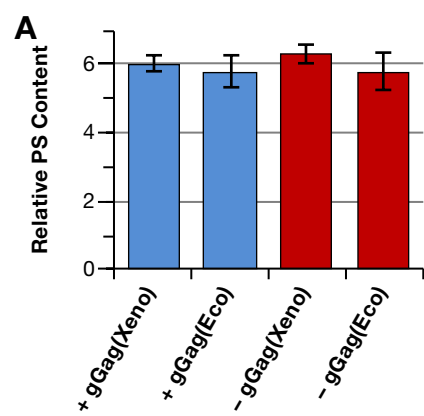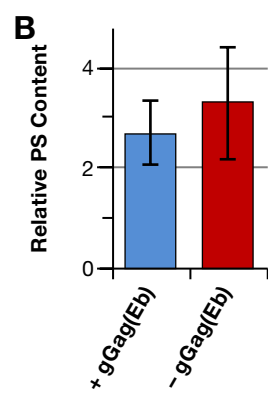

Supplement: Figure S2 — Glycogag does not affect annexin V binding to virions. Relative PS levels on MLV(Eco) and MLV(Xeno) (A) and MLV(Eb-FL) (B) produced using wild-type Gag-Pol (blue bars) or mutant Gag-Pol lacking gGag (red bars). The units of the relative values are arbitrary, and values cannot be compared between panels A and B. Download [file mbo006163085sf2.pdf]

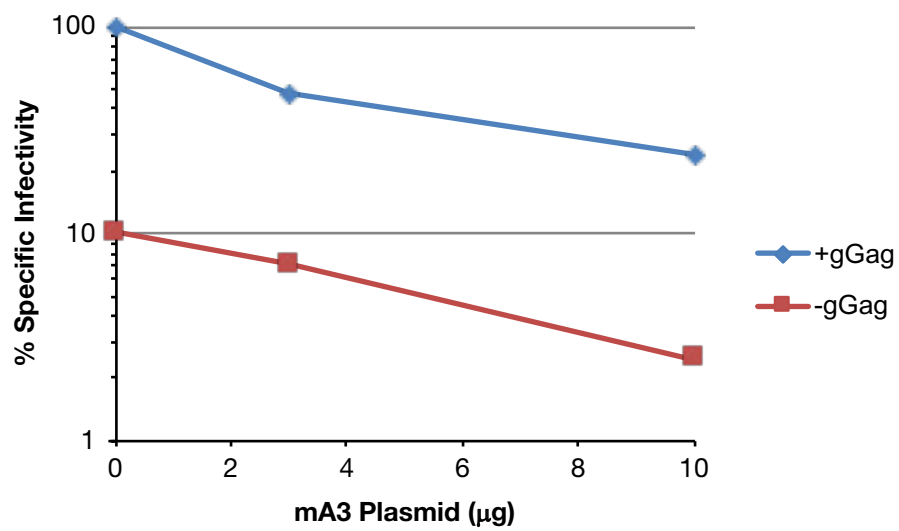

Supplement: Figure S3 — Glycogag does not affect sensitivity of MLV(Xeno) to mA3. Specific infectivity of MLV(Xeno) produced with wild-type Gag-Pol (blue line) or with the mutant Gag-Pol lacking gGag (red line) by transient transfection of 293T cells in the presence of increasing amounts of mA3 expression plasmid. Download [file mbo006163085sf3.pdf]

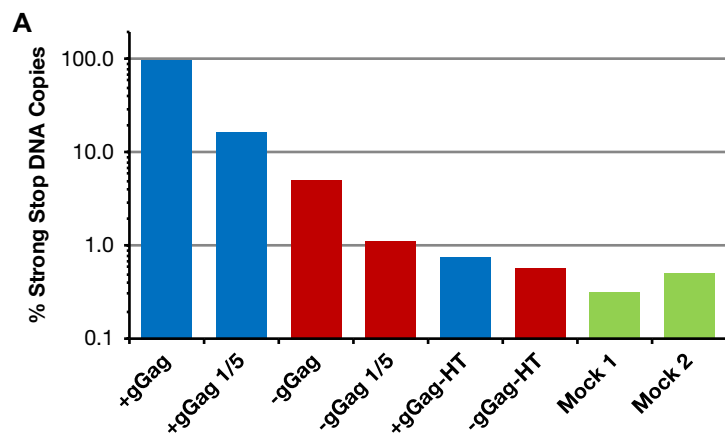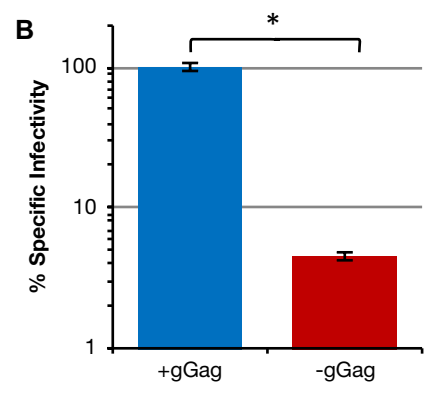

Supplement: Figure S4 — Viral DNA synthesis by MLV(Xeno) with or without glycogag. (A) MLV was produced by transfection of 293T cells with wild-type Gag-Pol (blue bars) or the mutant Gag-Pol lacking glycogag (red bars) together with the xenotropic Env expression plasmid. HT1080 cells were then infected with these viruses, either undiluted or diluted (1/5) or heat treatment inactivated (HT), or were mock infected. The cells were assayed 24 h later for MLV minus-strand strong-stop DNA. (B) Specific infectivities of viruses used in the experiment whose results are shown in panel A *, P < 0.0001. Download [file mbo006163085sf4.pdf]

**A**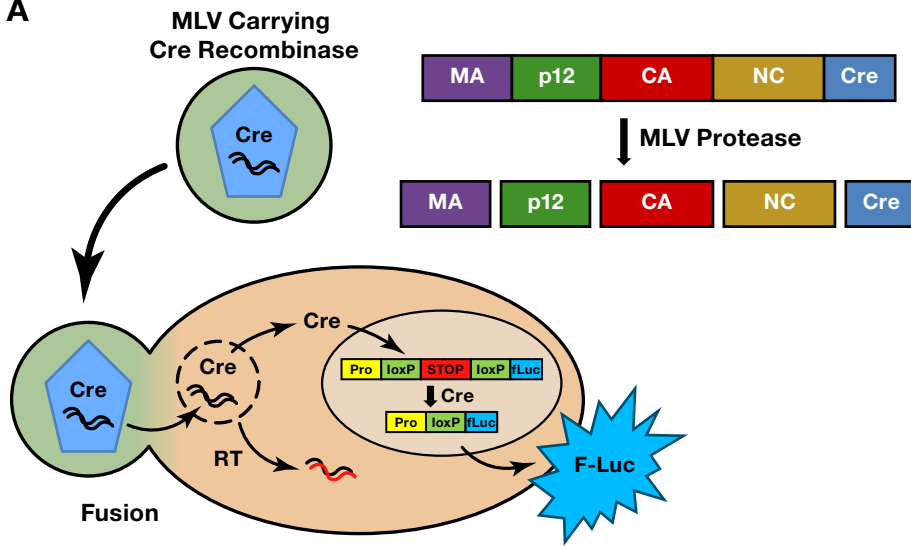**B**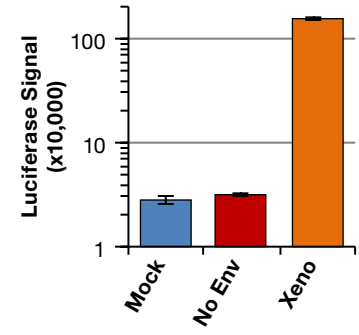

Supplement: Figure S5 — Strategy of MLV entry assay. (A) Schematic depiction of the entry assay. The inset shows the expected cleavage pattern of the Gag-Cre protein. (B) Signal in the luciferase assay performed on Cre reporter cells at 48 h postinfection. The cells were mock infected or infected with MLV carrying Cre recombinase, either with no Env (red bar) or with Xeno Env (orange bar). Counts obtained in the luciferase assay measured in triplicate ± standard deviation are shown. Download [file mbo006163085sf5.pdf]

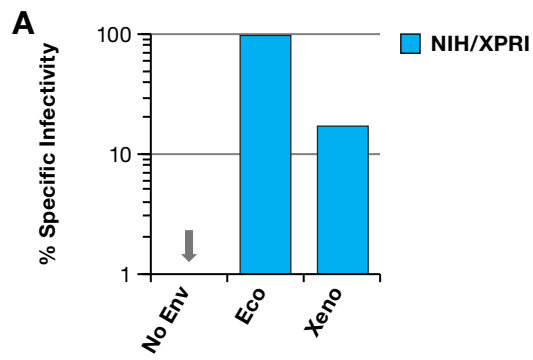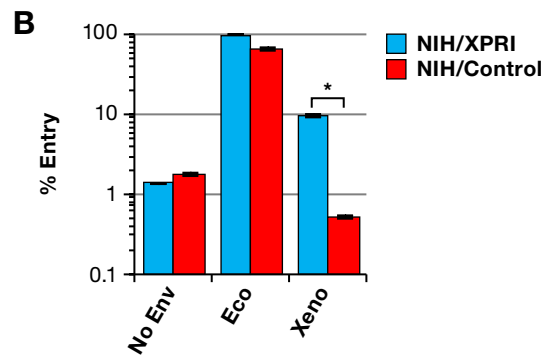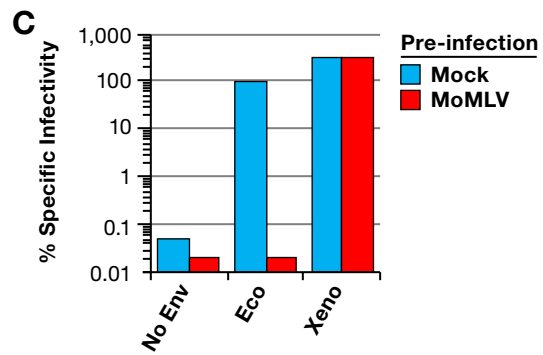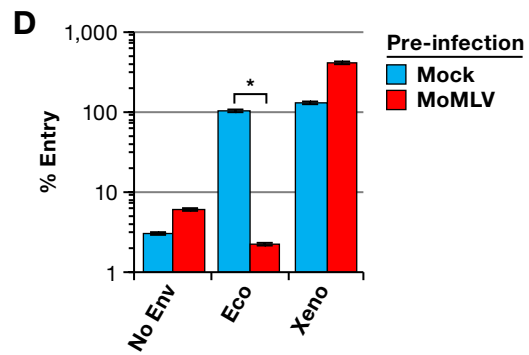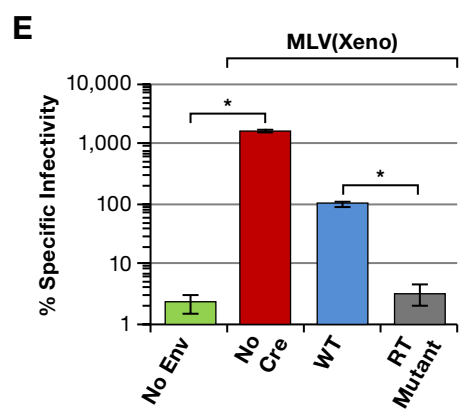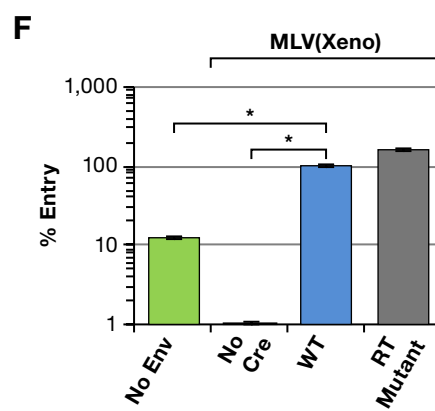

Supplement: Figure S6 — Validation of MLV entry assay. (A and B) Specific infectivities (A) and entry (B) of MLV with wild-type Gag-Pol, produced with no envelope (No Env) or Eco or Xeno envelope, assayed on NIH/control or NIH/XPR1 cells which had been transiently transfected with the Cre reporter cassette. (C and D) Cre reporter cells were pre-infected with MoMLV or were mock-infected and passaged for 2 weeks. The viruses used in the experiments whose results are shown in panels A and B were then assayed for specific infectivity (C) and entry (D) on these cells. In the specific infectivity graphs, the bars represent the percentage of GFP-positive cells normalized to the amount of virus in the samples. The entry and specific infectivity values are the percentages of the values for MLV(Eco). (E and F) Specific infectivity (E) and entry (F) of MLV with wild-type (blue bar) or DD224-225AA RT mutant (grey bar) Gag-Pol carrying Cre recombinase and Xeno Env, assayed on the Cre reporter cells. No Env, MLV with wild-type Gag-Pol but no Env glycoprotein; No Cre, MLV with wild-type Gag-Pol and Xeno Env but without Cre recombinase. *, P < 0.0001. Download [file mbo006163085sf6.pdf]
